# The role of social media messages and content creators in shaping COVID-19 vaccination intentions

**DOI:** 10.3389/fdgth.2025.1448884

**Published:** 2025-03-24

**Authors:** Xiaofeng Jia, Soyeon Ahn, Susan E. Morgan

**Affiliations:** ^1^ School of Media & Communication, Bowling Green State University, Bowling Green, OH, United States; ^2^ School of Education and Human Development, University of Miami, Miami, FL, United States; ^3^ School of Communication, University of Miami, Miami, FL, United States

**Keywords:** vaccination intention, social media, message strategy, user engagement, health behavior

## Abstract

**Introduction:**

Social media plays a crucial role in shaping health behaviors by influencing users' perceptions and engagement with health-related content. Understanding these dynamics is important as new social media technologies and changing health behaviors shape how people engage with health messages.

**Aim:**

The current study explored the relationship between the characteristics of content creators, the messaging strategies employed in social media, and users' engagement with social media content, and whether these features are linked to users' behavioral intentions.

**Methods:**

This study adopts a cross-sectional survey design. A total of 1,141 participants were recruited. We have developed a structural equation model to investigate the relationships between the characteristics of content creators, the messaging strategies employed in social media, users’ perceived HBM constructs, user engagement, and users' behavioral intentions.

**Results:**

Results revealed that social media posts focusing on self-efficacy were linked to increased willingness to engage in healthy behaviors. Additionally, individuals who demonstrate stronger perceptions of HBM constructs—such as higher perceived susceptibility and benefits of vaccination—are more likely to engage with posts, which was associated with higher vaccination intention. Posts authored by celebrities garnered a relatively higher number of favorites, while a greater proportion of politicians as content creators was linked to increased user comment intention.

**Conclusion:**

Our study underscores the potential of integrating the Health Belief Model into social media to help promote health behaviors like the COVID-19 vaccination. Furthermore, our findings offer valuable insights for professionals and policymakers, guiding them in crafting effective message strategies and selecting appropriate sources to promote health behaviors on social media platforms.

## 1 Introduction

The volume of information disseminated on social media has significantly expanded, encompassing scientifically valid data and evidence-based recommendations along with low-quality data, personal opinions, disinformation, and misinformation (1). Beyond the content itself, the effects of social media are increased by its unique features, such as the opportunity for user engagement (2). User engagement involves a state of cognitive and emotional absorption and is determined by social media activities such as searching for, viewing, commenting on, and even sharing social media content online (3). These types of engagement are consequential; Alhabash et al. (4) demonstrated that social media users are more likely to engage in offline behaviors if they receive persuasive messages and respond positively.

Social media platforms influence how billions of people act and think every day, so we need to know how the content and the creators of social media messages affect users' engagement and real-world actions. For example, during the pandemic, social media has played an important role in disseminating COVID-19 vaccine information, much of which had a positive impact. A cross-sectional social media-based survey conducted in the USA in 2021 showed that 81.5% of social media users had a positive attitude toward COVID-19 vaccination, with 91.9% considering it an act of civic responsibility (5). Xin et al. (6) discovered that frequent social media exposure and interpersonal discussion were positively associated with COVID-19 vaccination intention among specific populations, like nurses.

Despite these insights, existing research has primarily focused on the general impact of social media on vaccine attitudes and behaviors, often without systematically examining the specific characteristics of content creators and message strategies that drive engagement. This gap is particularly relevant given the increasing role of social media in shaping public health discourse, yet little is known about which factors most effectively enhance engagement with health-related messages.

Failing to address this gap poses a risk to public health communication efforts, as ineffective messaging strategies may lead to disengagement, misinformation spread, or even public resistance to critical health initiatives. Without empirical insights into what makes health-related content more engaging, public health agencies may struggle to design campaigns that effectively counter misinformation and encourage positive health behaviors. Therefore, it is important for us to understand how source and message characteristics affect the impact to design effective health communication strategies and interventions on social media.

The current study aims to examine the relationship between the characteristics of content creators, the messaging strategies employed in social media, and users' engagement with social media content, and whether these features are linked to users' behavioral intentions, using a COVID-19 vaccination as an example. Our study helps to understand how users of social media platforms understand messages related to critical health issues. We hope that our research will offer the basis for future research and practice, by using the insights we gain from it. Specifically, our study will help governmental and health professionals to create social media campaigns that encourage desired health behaviors in a systematic way. Also, this study will help researchers develop empirical models that might explain user engagement better within social media platforms.

## 2 Literature review and hypothesis development

### 2.1 The role of social media on health behaviors

Social media comprises internet-based applications that empower users to establish virtual networks and communities. It serves as a platform for interaction, enabling users to share their ideas, thoughts, and information in diverse formats, including text, pictures, videos, and status updates (7–9). In today's digital landscape, social media stands out as a dynamic and rapidly expanding resource. It encompasses a diverse array of platforms and applications with national, regional and global reach that cater to users of all ages (10, 11). Users can leverage social media platforms to connect and engage with friends and like-minded individuals who share common interests (12, 13). Moreover, social media provides individuals with the opportunity to access knowledge and insights, offering a window into various opinions and perspectives on a wide range of topics, issues, and events (14, 15).

Social media holds the potential to exert a positive impact on health attitudes and behaviors, including fostering favorable attitudes towards vaccines. Many studies underscore this impact. For example, Melton et al. (16) documented an enduring surge in positive sentiments within Reddit communities when discussing the COVID-19 vaccine, reflecting shifts in affective attitudes. Likewise, Liu (17) established a clear positive correlation between engagement with COVID-19 information on social media and the adoption of preventive behaviors. Similarly, Biella et al. (18) found that frequent exposure to pro-vaccine content on social media was associated with reduced susceptibility to anti-vaccination attitudes, which are key predictors of lower vaccine intentions.

However, the widespread presence of anti-vaccination communities on social media has also deepened polarization, reinforcing skepticism and misinformation. Prior work has shown that anti-vaccination attitudes are closely linked to lower intentions to perform vaccination-related behaviors, such as getting vaccinated (19). Furthermore, misinformation on social media can influence vaccine policy debates, as seen in cases where viral narratives shaped public opposition to vaccine mandates (20). In addition, the overwhelming volume of social media posts can contribute to message fatigue and information overload, potentially diminishing users' engagement and responsiveness to health messages (21–23).

This dual role of social media—both promoting and hindering vaccine uptake—underscores the need for a deeper understanding of how social media shapes health intentions and behaviors. By expanding on existing research, this study aims to offer insights that can help decision-makers strategically leverage social media to promote public health initiatives, with COVID-19 vaccination serving as a critical case study.

### 2.2 Health belief model and health behaviors

The Health Belief Model (HBM) is one of the most frequently employed theoretical frameworks used to explain health behaviors across a wide variety of contexts and target populations. It has been instrumental in shaping interventions aimed at promoting healthy behaviors such as vaccination and disease prevention. The HBM posits that an individual's health-related behavior is determined by their perception of the seriousness and likelihood of a health threat, as well as the perceived advantages and hindrances associated with the adoption of a recommended health action (24).

The HBM comprises six key components: perceived susceptibility, perceived severity, perceived benefits, perceived barriers, cues to action, and self-efficacy. Essentially, the HBM centers around individuals' perceptions of threat and their assessment of suggested behaviors. Threat perception is divided into two beliefs: the belief in one's susceptibility to a particular health issue and the belief in the severity of the consequences of that illness. Evaluation of the recommended behavior also encompasses two separate sets of beliefs: considerations regarding the benefits or effectiveness of the suggested health behavior and considerations regarding the obstacles to adopting the health behavior. Cues to action are the stimulus needed to trigger the decision-making process to adopt a recommended health action (24). These cues can be internal or external. Individuals' perceptions of symptoms (e.g., chest pains, wheezing, etc.) are examples of internal cues to action (25). External cues include advice from others, the illness of a family member or friend, social media campaigns, or healthcare providers promoting health-related behaviors (25). Self-efficacy pertains to the degree of an individual's assurance in their capacity to carry out a behavior, such as getting vaccinated. The value of a recommendation for a health behavior relies on whether one feels capable of competently executing the necessary steps to engage in the behavior (26).

Multiple reviews demonstrated HBM's effectiveness in predicting and explaining preventive health behaviors (25, 27–30). Particularly, perceived benefits and barriers consistently emerged as the most robust predictors, while perceived susceptibility and severity either exhibit weak associations with preventive health behaviors or fail to demonstrate statistically significant links to behavior change (25). Despite variations in the effectiveness of individual HBM constructs, the model continues to serve as a foundational theoretical basis for numerous interventions and prevention programs (31).

Recent studies, particularly those focused on COVID-19 vaccination, have reaffirmed the predictive power of HBM constructs. Specifically, perceived susceptibility, perceived severity, perceived benefits, perceived barriers, cue to action, and self-efficacy have all been identified as predictors of COVID-19 vaccination willingness, intentions, and behaviors (32–35). Furthermore, perceived benefits and perceived barriers have been shown to impact vaccine intentions significantly and directly. These findings inform the first hypothesis of the current study.

***H1***: Social media users holding higher levels of perceived severity, susceptibility, benefits, barriers, self-efficacy, and cues to actions related to COVID-19 vaccination will be more likely to have higher levels of people's intentions to receive the COVID-19 vaccine (*H1a*) and intentions to persuade others to take the vaccine (*H1b*).

While perceived HBM constructs reflect an individual's personal beliefs about health behaviors (e.g., their perception of COVID-19 severity or vaccine benefits), HBM constructs are also commonly incorporated into social media posts to frame health messages. HBM message constructs refer to how these concepts are framed within social media content (e.g., whether a post highlights the severity of COVID-19 or emphasizes the benefits of vaccination). All six HBM constructs—severity, susceptibility, benefits, barriers, self-efficacy, and cues to actions, appear in social media messages to some extent. Previous Research suggests that social media messages incorporating HBM constructs can influence user engagement and persuasive outcomes (36).

However, the frequency of using each construct in social media messages depends on the health topic. For example, the benefit of the COVID-19 vaccine was mentioned frequently in a study assessing public health social media messages targeting COVID-19 prevention behaviors disseminated by governmental agencies (37). Another study analyzing content related to healthy dietary practices on YouTube found that HBM message constructs were rarely used for this topic (38). However, posts that did use HBM message constructs received significantly more favorites, comments, and shares. Given that exposure to certain message frames may activate or reinforce users' existing HBM perceptions, it is essential to examine how social media messages utilizing these constructs relate to vaccine-related intentions. These findings inform the second hypothesis of the current study.

***H2*:** Social media messages that have higher levels of HBM message constructs—severity, susceptibility, benefits, barriers, and self-efficacy about COVID19 vaccine—will be linked significantly with vaccination intentions (*H2a*) and intentions to persuade others to take the vaccine (*H2b*).

### 2.3 Characteristics of content creators and health outcomes

The features of content creators can also influence the audience's behaviors (39). Recent research has shown that leveraging the power of social media influencers has led to favorable health outcomes in various areas, including HPV vaccination, tobacco prevention, healthy eating, and skin cancer prevention (40). Also, previous research has shown that factors such as gender (41) and occupation (42, 43) of social media content creators can influence the health behaviors of various targeted audiences. However, the effects of other personal traits of content creators, such as age, race, and occupation on people's health behaviors have not been fully explored yet.

While individual characteristics influence audience perceptions across various media formats, social media presents unique affordances that distinguish it from traditional media like TV and radio. Unlike passive media consumption, social media fosters real-time engagement, allowing audiences to interact with content creators through comments, likes, and shares, which can heighten perceived credibility and influence (44). Moreover, the visibility of creators' personal traits—such as gender, age, and occupation—is often more pronounced on social media due to profile accessibility and engagement metrics, making measurement more convenient. While the effects of certain creator traits (e.g., gender and occupation) on health behaviors have been explored, the influence of other personal characteristics, such as age and race, remains under-examined. Understanding how these characteristics influence vaccination intentions and health behaviors in social media contexts is one of the objectives of this study.

***RQ1*:** Are there significant associations between content creators' personal traits (i.e., gender, race, and occupation) and outcome variables (vaccination intentions and intentions to persuade others to take the vaccine)?

### 2.4 Social media user engagement

User engagement is defined as a category of user experience that is characterized by a state of cognitive and emotional absorption (45). Studies have consistently shown that when users respond positively to persuasive messages shared on social media, they are more likely to perform specific offline actions, such as purchasing (4). In the context of health communication, research indicates that engaging in activities on social media helps users maintain health-related behaviors, including physical activity and making healthy dietary choices (46, 47), endorsing e-cigarette policies (48), and influencing the clinical outcomes of individuals with diabetes (49).

Various social media engagement behaviors have distinct qualitative characteristics. First, the act of “Liking” a post represents the simplest form of engagement, involving no verbal expression. Research consistently points to a positive association between “liking” and the development of favorable attitudes and behavior change. Second, “sharing” content is a higher level of engagement, effectively transforming users into voluntary messengers with the potential to influence purchasing decisions or health behaviors. Third, “commenting” is considered the most substantial form of engagement, demanding more time and effort, thereby allowing users to express opinions and share information directly with others (4, 50–52).

Nevertheless, our understanding of how different social media user engagement behaviors interact with message and source characteristics in social media posts and their impact on users' health outcomes is limited. This literature informs the following two research questions of the current study.

***RQ2*:** Are there significant associations between the independent variables (users' perceived levels of HBM related to COVID-19 vaccination, HBM message constructs, content creators' characteristics) and user engagement variables (favorites, comments, and shares)?

***RQ3*:** Are the relationships between the independent variables (users' perceived levels of HBM related to COVID-19 vaccination, HBM message constructs, content creators' characteristics) and outcome variables (vaccination intentions and intention to persuade others to take the vaccine) mediated by user engagement variables (favorites, comments, and shares)?

**Figure 1:**
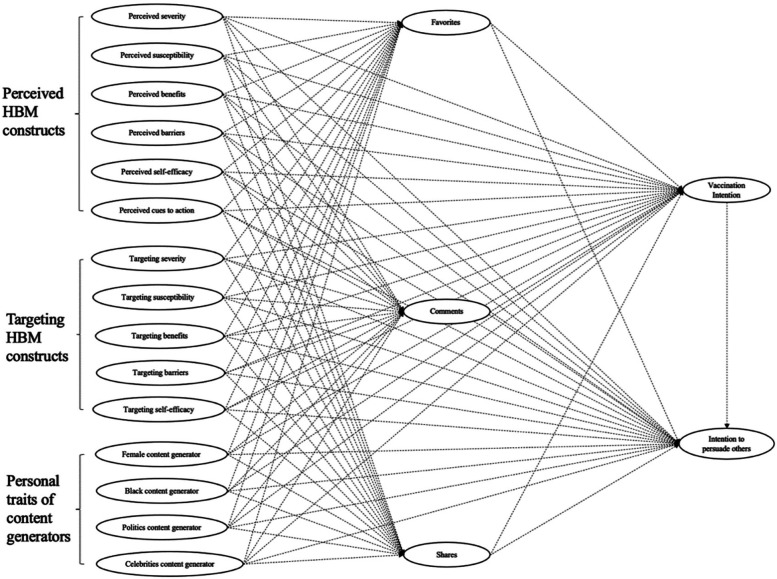
The conceptual model.

Based on the theoretical framework and research objectives, our conceptual model is shown in Figure 1.

## 3 Methods

### 3.1 Sample

The target population for this study is all adults who engage with social media platforms, have not been fully vaccinated, and have not formed an intention to get vaccinated. In order to make inferences about this target population, the sample inclusion criteria were set as follows: (1) an individual must be over 18 years old, (2) an individual must have used one or more mainstream social media platforms at least once in the last year, and (3) an individual must not have been fully vaccinated; in other words, participants may not have received two or more doses of COVID-19 vaccines to participate in the study. Additionally, participants who failed to pass the attention check question were excluded. Amazon Mechanical Turk (MTurk) was used to recruit participants for our survey, which was administered through Qualtrics. MTurk was selected because it provides a diverse and relatively representative sample of the general population, allows for efficient and cost-effective data collection, and has been widely used in social science research. Data collected on MTurk has been proven to be as reliable as those obtained from traditional survey methods (53–55).

Initially, 2,331 participants accessed the survey, of which 1,141 individuals met the inclusion criteria and were included in the final sample. Among these participants, 45.0% (*n* = 514) were female, 82.7% identified as White (*n* = 944), and 33.6% identified as Hispanic (*n* = 383). Participants' ages ranged from 18 to 79 (*M* = 33.82, *SD* = 10.81). The majority (93.9%) had obtained at least an undergraduate degree, and 33.9% reported a household income of $75,000 or more in the previous year (which is higher than the median US household income in 2021, $70,784). Detailed demographic information is presented in Table 1.

**Table 1:** Demographics of participants.

Demographic	Number (%) (*n* = 1,141)
Gender	
Female	514 (45.0%)
Male	627 (55.0%)
Race	
White	944 (82.7%)
American Indian or Alaska Native	30 (2.6%)
Asian	112 (9.8%)
Black or African	45 (3.9%)
American Native	5 (0.4%)
Hawaiian or Other Pacific Islander	1 (0.1%)
Others	3 (0.3%)
Prefer not to disclose	1 (0.1%)
Ethnicity	
Hispanic or Latino	383 (33.6%)
Not Hispanic or Latino	749 (65.6%)
Prefer not to disclose	9 (0.8%)
Education	
Some high school	1 (0.1%)
High school diploma	46 (4.0%)
Some college	23 (2.0%)
Bachelor's degree	809 (70.9%)
Master's degree or above	262 (23.0%)
Income	
Less than $20,000	36 (3.2%)
$20,000–$34,999	104 (9.1%)
$35,000–$49,999	229 (20.1%)
$50,000–$74,999	385 (33.7%)
$75,000–$99,999	289 (25.3%)
$100,000–$149,999	84 (7.4%)
$150,000 or more	14 (1.2%)
Age	*M* = 33.21, *SD* = 10.81

### 3.2 Stimuli

Social media posts and creator profiles were sourced from a publicly available dataset called “CoVaxxy.” The CoVaxxy dataset was created to explore vaccine hesitancy and its relationship to public health outcomes, focusing on a set of Twitter posts related to the COVID-19 vaccine (56). Because the current study aimed at identifying social media posts’ impact on positive attitudes and intentions toward COVID-19 vaccines, posts with anti-vaccine contents were omitted from the dataset. Next, social media posts were classified based on the message strategies used to support positive intentions. Tweets were not limited to using just one strategy. For instance, if a tweet emphasized the risk to a child of contracting COVID-19 but also mentioned the benefits of vaccination, it fell under the category of susceptibility in social media messages. 10 posts with higher user engagement were selected from each category, resulting in a stimulus pool of 50 tweets.

We selected 25 content creators' profiles to represent a balanced range of demographic characteristics, such as gender, race, and occupation. Each profile was paired with two tweets from different categories, both authored by the same content creator. We paired the posts using two key criteria: (1) balancing content creators by gender, race, and occupation for each strategy across ten posts, and (2) aligning the characteristics of the content creator with the content of the tweet. For example, in the “severity” category, there were five tweets from women, four from men, and one from an organization. Regarding race, there were six posts from White creators and three from Black creators, with two posts from each occupation category (e.g., politicians, health professionals, celebrities, writers/journalists, laypersons). Similarly, tweets originating from the White House needed to maintain a tone that was appropriate to the message. Two posts from each of the five strategy categories, with a total of ten social media posts, were randomly displayed to participants.

### 3.3 Data collection

Participants were presented with a consent form on MTurk before responding to survey questions. Upon providing consent to participate in the study, they were then directed to the online questionnaire. Participants first answered the screening questions about their ages, how often they used social media, and how many doses of the COVID-19 vaccine they received. Participants who did not meet the inclusion criteria were included in this step. Then, participants answered questions about how their perception of the COVID-19 vaccine, in order to measure their levels of perceived severity, susceptibility, benefits, barriers, self-efficacy, and cues to action. Next, participants were then randomly presented with 10 social media posts, each featuring creators' profiles, sourced from a publicly available dataset comprising COVID-19 vaccine-related tweets. Following each post, participants were asked to indicate how they would engage with each post, indicating their likelihood of “liking,” “sharing,” and “commenting” on the content. Subsequently, participants responded to questions about their intention to receive the COVID-19 vaccine, followed by inquiries about demographics, including age, gender, race, education level, and income.

### 3.4 Variables and measures

*Constructs of HBM.* A total of forty-two items correspond to the six constructs of the HBM model. The measures were adopted from previous studies using HBM in the context of the COVID-19 vaccine (33, 57–60). Six dimensions of health beliefs in the HBM were measured on a 5-point Likert scale, 1 being “strongly disagree” and 5 being “strongly agree”. A higher score indicates higher agreement on the statement regarding each health belief. These six constructs include:

(1) *Perceived susceptibility.* Three items measuring *perceived susceptibility* (Cronbach's *a* = .65, *M* = 3.85, *SD* = .73) to COVID-19 infection (e.g., I am at higher risk getting COVID-19),
(2) *Perceived severity.* Four items measuring *perceived severity* (Cronbach's *a* = .71, *M* = 4.02, *SD* = .60) of COVID-19 infection (e.g., If I got COVID-19, it would probably be more serious than the flu),
(3) *Perceived benefits.* Ten items measuring *perceived benefits* (Cronbach's *a* = .86, *M* = 3.98, *SD* = .56) of COVID-19 vaccination (e.g., Getting COVID-19 vaccine can reduce the chance of infection),
(4) *Perceived barriers.* Ten items measuring *perceived barriers* (Cronbach's *a* = .85, *M* = 3.86, *SD* = .62) to COVID-19 vaccination (e.g., I am concerned about the safety of COVID-19 vaccine),
(5) *Self-efficacy.* Five items measuring *self-efficacy* (Cronbach's *a* = .65, *M* = 4.03, *SD* = .53) (e.g., It is easy for me to get the COVID-19 vaccine),
(6) *Cues to action.* Ten items measuring *cues to action* (Cronbach's *a* = .87, *M* = 3.97, *SD* = .57) (e.g., If someone I like on social media encouraged COVID-19 vaccination, I would probably get the vaccine).

Because each social media post contains different HBM message constructs, these constructs in the post were dummy-coded. If the HBM construct appeared in the post, it was coded as 1; otherwise, it was coded as 0. For example, if a social media post targets users' perceived severity of COVID-19 and perceived barriers to the vaccine, the items “severity” and “barriers” were coded as 1, and the variables of message susceptibility, benefits, and self-efficacy were coded as 0. The “cues to action” variable was excluded when coding HBM message constructs in social media posts due to its reliance on the audience's perception and cannot be coded in these social media posts (36).

Since each participant was exposed to 10 social media posts, we calculated the frequency of each of the five message strategies across those posts, resulting in five continuous variables. For example, if the construct benefits were represented in 5 posts out of 10 posts, the sum of message benefits would be 5. This recoding process led to five new continuous variables: (1) the sum of the message severity score (*M* = 3.40, *SD* = 0.87), (2) the sum of the message susceptibility score (*M* = 2.40, *SD* = 0.54), (3) the sum of the message benefits score (*M* = 5.20, *SD* = 1.12), (4) the sum of message barriers score (*M* = 4.60, *SD* = 1.19), and (5) the sum of message self-efficacy score (*M* = 2.60, *SD* = 0.65).

*User Engagement Intentions.* User engagement intentions were assessed using three questions: “How likely is it that you would ‘*like*’ this post?” (*M* = 3.96, *SD* = 0.63) “How likely is it that you would ‘*share*’ this post?” (*M* = 4.02, *SD* = 0.71) and “How likely is that you would make a *comment* on this post?” (*M* = 3.93, *SD* = 0.63). The measurement was adopted from Ibrahim et al. (61). User engagement intentions were measured with a 5-point Likert scale (1—Extremely unlikely and 5—Extremely likely). A higher score indicates participants are more likely to engage with social media posts.

*Intentions to Vaccinate.* Intentions to vaccinate were assessed using “*If you were offered a COVID-19 vaccine or booster shot for free, what would you do?*” (*M* = 4.02, *SD* = 1.05) on a 5-point Likert scale (62). A higher score indicates participants are more likely to get vaccinated.

*Support of Vaccination.* Support of vaccination was assessed using “*If your family members wanted to get the COVID-19 shot or booster shot, what would you do?”* (*M* = 3.59, *SD* = 1.13) on a 5-point Likert scale (62). A higher score indicates participants have intentions to be more supportive of the COVID-19 vaccination.

*Demographics.* Participants' demographics include gender (i.e., female, male, non-binary/third gender, and prefer not to disclose), age (i.e., on a continuum from 18 to 100), race (i.e., White, American Indian or Alaska Native, Asian, Black or African, American Native, Hawaiian or Other Pacific Islander, other races, and prefer not to disclose), ethnicity (i.e., Hispanic or Latino, non-Hispanic or Latino, and prefer not to disclose), educational level (i.e., some high school, high school diploma, some college/no degree, bachelor's degree, or master's degree and above), and household income level (i.e., less than $20,000; $20,000–$34,999; $35,000–$49,999; $50,000–$74,999; $75,000–$99,999; $100,000–$149,999; and $150,000 or more).

### 3.5 Priori- and posterior power analysis

*A priori* power analysis was conducted following the approach described by Preacher Coffman (63) to find out the minimum sample size required for the desired RMSEA level. The results indicate that at least 186 participants are needed to reject the null hypothesis of RMSEA greater than or equal to .05 in favor of close fit, with a statistical power of .90 when *df* = 172, alpha is .05. Thus, the obtained sample size of *N* = 1,141 is adequate to test the study hypothesis. In addition, the posterior power analysis results indicated that the power reaches 1, when *n* = 1,141, *df* = 172, and RMSEA = .041.

### 3.6 Data analysis

A confirmatory factor analysis (CFA) was performed to examine the underlying measurement model. When the measurement model was considered as an acceptable fit, structural equation modeling (SEM) was conducted using *Mplus* (version 7.4 (64); to explore hypotheses presented in Figure 1. Overall model fit was assessed using (1) Comparative Fit Index (CFI) or Tucker–Lewis Index (TLI) ≥ .90, Standardized Root Mean Square Residual (SRMR) ≤ .10, or (2) Root Mean Square Error of Approximation (RMSEA) ≤ .08. The significance of each path was then examined at an alpha level of.05. For testing indirect-effects, a bias-corrected bootstrapping procedure (bootstrapped sample size = 5,000) with 95% confidence intervals was implemented to conduct the mediation analyses. This approach is more rigorous for examining the proposed mediation effects because the indirect effect in SEM using bias-corrected bootstrapped samples employs a full-information Maximum Likelihood technique. The indirect effect was considered significant when the bias-corrected bootstrapping confidence intervals did not include zero.

## 4 Results

### 4.1 Measurement model

Results from the initial measurement model demonstrated a poor fit to the data [*χ*^2^(804) = 5,803.15, *p* < 0.05, RMSEA = 0.074 (90% CI = 0.07–0.08), CFI = 0.77, TLI = 0.75, SRMR = 0.06]. Upon allowing the correlations between items under the same construct, the model fit improved substantially [*χ*^2^(743) = 3,819.37, *p* < 0.05, RMSEA = 0.06 (90% CI = 0.058, 0.062), CFI = 0.86, TLI = 0.83, SRMR = 0.06]. The standardized factor loadings were from 0.60 to 0.74 (perceived severity); 0.53 to 0.61 (perceived susceptibility); 0.50 to 0.71 (perceived benefits); 0.50 to 0.71 (perceived barriers), 0.46 to 0.57 (perceived self-efficacy), and 0.55 to 0.65 (perceived cues to action). All of the factor loadings were found to be statistically significant.

### 4.2 Structural model

**Figure 2:**
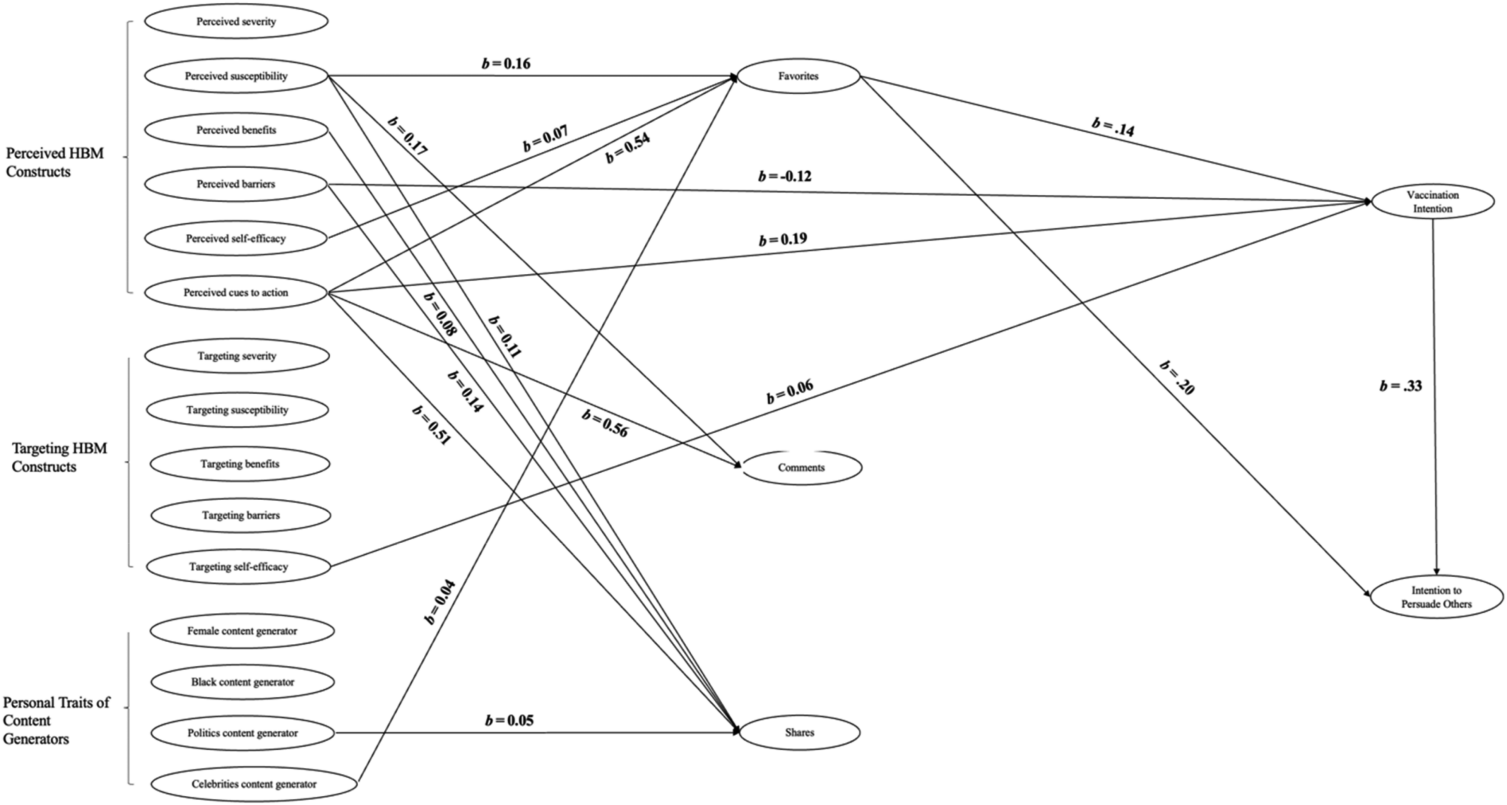
The final model (only significant paths are displayed).

Results from the structural model indicate an acceptable fit to the data [*χ*^2^(114) = 337.57, *p* < 0.05, RMSEA = 0.04 (90% CI = 0.04–0.05), CFI = 0.94, TLI = 0.92, SRMR = 0.06]. Figure 2 illustrates the outcomes of the structural model. The following section summarizes the findings for each hypothesis.

*Hypothesis 1–*For *H1a*, perceived barriers to getting the COVID-19 vaccine were negatively correlated with their vaccination intention (*b* = −0.12, *SE* = 0.05, *p* = 0.01). In addition, people's perceived cues to action were positively associated with their intentions to vaccinate (*b* = 0.19, *SE* = 0.06, *p* = 0.002). Others were not found to be statistically significant. *H1b* was not supported.

*Hypothesis 2–*For H2a, the results showed that when tweets contained content users' self-efficacy construct, the more that users intend to get vaccinated (*b* = 0.06, *SE* = 0.03, *p* = 0.04). Other HBM constructs were not related to vaccine intentions. *H2b* was not supported.

*Research Question 1– Content creator characteristics and vaccine intentions.* We did not find a significant relationship between content creators' personal traits and people's vaccine intentions.

*Research Question 2– Relationships between predictors and user engagement*. The results showed that social media users' perceived susceptibility to COVID-19 (*b* = 0.16, *SE* = 0.03, *p* < 0.001), self-efficacy to take the COVID-19 vaccine (*b* = 0.07, *SE* = 0.03, *p* = 0.04), and cues to action (*b* = 0.54, *SE* = 0.04, *p* < 0.001) were all positively and significantly associated with their liking of the posts. Additionally, perceived susceptibility to COVID-19 (*b* = 0.17, *SE* = 0.03, *p* < 0.001) and the perceived presence of cues to action (*b* = 0.56, *SE* = 0.038, *p* < 0.001) were both associated with participants' intentions to comment on the posts. Lastly, perceived susceptibility to COVID-19 (*b* = 0.11, *SE* = 0.029, *p* < 0.001), perceived benefits of getting the COVID-19 vaccine (*b* = 0.08, *SE* = 0.04, *p* = 0.043), barriers to taking the COVID-19 vaccine (*b* = 0.14, *SE* = 0.03, *p* < 0.001), and cues to action (*b* = 0.51, *SE* = 0.04, *p* < 0.001) lead to more sharing of the posts.

In terms of content creators' personal traits, the results showed that tweets from celebrities received significantly more favorites (*b* = 0.04, *SE* = 0.02, *p* = 0.05) than other types of occupations. Moreover, tweets by politicians received significantly more shares (*b* = 0.05, *SE* = 0.02, *p* = 0.03) than other occupations.

*Research Question 3- Indirect effects of user engagement*. The results showed that the paths from participants’ (a) perceived susceptibility [*b* = .03, *SE* = .01, *p* = .02, 95%CI (.01, .07)], and (b) perceived cues to action [*b* = .14, *SE* = .06, *p* = .01, 95%CI (.04, .26] to vaccination intention were significantly mediated by the number of favorites they gave to a tweet. In addition, the results showed that the paths from participants' (a) perceived susceptibility [*b* = .05, *SE* = .02, *p* = .003, 95%CI (.02, .09)], (b) perceived cues to action [*b* = .13, *SE* = .05, *p* = .01, 95%CI (.12, .34)] to the intentions to persuade others to get vaccines were significantly mediated by the number of favorites they gave. Figure 2 shows the final model (Only significant paths are displayed). The correlation matrix was provided in Appendix 1.

## 5 Discussion

### 5.1 Summary of findings

The current research examined whether perceived level of HBM constructs about COVID-19 vaccination, HBM constructs in social media posts, and the individual traits of content creators are related to (1) social media users' behavioral intentions; (2) intentions to persuade others to take vaccines; and (3) whether user engagement plays a mediating role of the relationships.

We found significant relationships between participants' exposure to various creator characteristics and their engagement with the presented stimuli, which is consistent with previous research on the topic of health promotion. Our findings are consistent with previous research that found that posts authored by celebrities garnered a relatively higher intention of “like” the post (65–67), underscoring the potential for greater celebrity involvement in public health promotion efforts. Furthermore, our analysis revealed a positive association between the proportion of content creators who are politicians and user comment intention. Commenting, as compared to liking and sharing, signifies the most engaged level of interaction, requiring users to invest more time and effort in providing direct responses to social media content (52). Social media users often utilize comments to express support or disagreement with the opinions of content creators, particularly on content related to the COVID-19 vaccine. Past studies have shown that politicians tend to adopt a celebrity-like style in their social media presence and marketing efforts (68, 69), actively engaging with users (70). While our findings shed light on the potential roles of celebrities and politicians in enhancing public understanding of health issues on social media, it is important to note that our study did not reveal a direct relationship between participants' exposure to different content creator characteristics and their vaccination intentions.

In addition to personal characteristics, our study showed that perceptions of several HBM constructs exhibited positive relationships with user engagement behaviors. HBM has been used widely in the development of health education messages and campaigns and in previous research, it also has been used to examine the content and engagement patterns associated with vaccine-promoting posts on social media (71, 72). For example, our results indicate that perceived susceptibility and perceived cues to action displayed positive correlations with all three types of engagement behaviors. These findings suggest that individuals who believed they were at higher risk of contracting COVID-19 were more likely to engage with social media posts related to the COVID-19 vaccine. Similarly, people who encountered stimuli that triggered the decision-making process, such as experiencing symptoms of the disease or receiving advice from others, also exhibited a greater intention to engage with social media content promoting the vaccine. In addition, our findings demonstrated a positive relationship between individuals perceived self-efficacy in getting vaccinated and the intention to “like” posts promoting the vaccine. “Liking” is the easiest form of engagement behavior on social media requiring no verbal expression but signifying support and agreement with the content's viewpoints (50). This study indicates that people who have higher self-efficacy regarding the COVID-19 vaccine are more inclined to use the “like” function to express support for posts endorsing the vaccine.

This study also examined the effect of perceived benefits and barriers associated with COVID-19 vaccination. Greater perceptions of the benefits and lower barriers of COVID-19 vaccination were positively correlated with individuals' intention to share the posts, which required a higher level of user engagement compared to liking (4). This implies that groups who perceive greater benefits or fewer barriers to the COVID-19 vaccines are more likely to participate in discussions about vaccines on social media. By implication, they may be more willing to defend their beliefs and to oppose contrary views. These findings contribute to the body of evidence about the relation between the messaging strategies that are used in social media posts promoting the COVID-19 vaccine and user engagement behaviors through the lens of HBM.

Perhaps more importantly, the objective of the current research was to examine whether participants' exposure to different features of social media content is associated with their health behavioral intentions. The results supported the utility of the HBM framework within the context of COVID-19 vaccination. This study found people who perceive greater barriers to getting the COVID-19 vaccine have a relatively lower intention to get the vaccine. In addition, individuals' perceived cues to action also had a positive relationship with their vaccination intention, which was also consistent with previous studies (62, 73–76). In addition, content that emphasized self-efficacy was positively associated with users' vaccination intention; this appears to be the most effective way to increase the audience's vaccination intention. These findings are consistent with previous research that showed that self-efficacy is a direct predictor of whether a person performs the desired behavior, including the willingness to get the COVID-19 vaccine (33, 77). Specifically, Guidry and colleagues' research (72) suggested that health professionals should focus social media posts in such a way as to increase perceptions of the self-efficacy of protective behavior. Based on our findings, we echo these recommendations; among the HBM constructs, self-efficacy has the strongest positive association with social media users' intention to get vaccinated. This information can be useful for public health professionals and decision-makers in their efforts to promote vaccine uptake.

In addition to the HBM constructs, we found that the number of “favorites” played a pivotal mediating role between perceptions of HBM constructs and individuals’ behavioral intentions. Specifically, the links between (a) perceived susceptibility (b) perceived cues to action, and (c) vaccination intention as well as (d) the intention to persuade others to get vaccines were fully mediated by the number of favorites the participant gave to social media posts. While prior research on engagement has closely examined the behaviors of social media users in health-promoting contexts (78–80), it remained unclear which forms of engagement behaviors were predictive of changes in health behaviors. The present study provides insights into how different types of user engagement behaviors influence vaccination intentions. Among them, we found that “favorites” significantly contribute to the relationship between individuals' perceptions of the COVID-19 vaccine and their vaccination intentions. These findings enhance our understanding of how individuals form opinions and make decisions regarding vaccination, offering valuable insights into the influence of diverse types of engagement behaviors on these choices.

### 5.2 Implications

Our study offers valuable insights into how the HBM can serve as a theoretical framework for designing and implementing social media messages and interventions. Our approach maintains ecological validity by presenting participants with real social media content. This approach makes our study more practical, as it shows how HBM concepts influence engagement intentions in response to authentic content. Additionally, the research adds to the body of evidence regarding the relationships between messaging strategies used to promote health behaviors, user engagement behaviors, and vaccination intentions from the perspective of the HBM. Lastly, the study advances our understanding of how individuals form opinions and make decisions about vaccination when exposed to information on social media. Moreover, the research scrutinizes the interplay of HBM constructs within social media messages, providing insights into the relative importance of different HBM factors in shaping users' decisions. Finally, the study illuminates the impact of different types of engagement behaviors in shaping vaccination decisions, contributing to a more nuanced understanding of how social media can effectively promote public health behaviors.

Our study can help public health professionals understand what factors affect vaccine acceptance among people and how to use them in their campaigns through social media platforms. Also, opinion leaders and influencers on social media with these factors can share accurate information and motivate vaccination among their followers. Moreover, health campaign designers can learn the best message strategies for social media posts and how to improve the quality of their content. Lastly, it helps public health experts to create effective interventions and campaigns to promote vaccination on social media. In short, the current research provides useful insights to help public health professionals use evidence-based strategies to increase vaccine uptake and support positive health behaviors on social media.

### 5.3 Limitations and conclusion

There are some limitations of this study that warrant attention. First, the results of this study rely on correlational data, which imposes some constraints on the insights it can offer. To establish possible causal relationships between the elements in support-seeking messages and social support behaviors, a controlled experimental study may be necessary.

Another limitation pertains to the choice of prioritizing external validity over internal control. In the current study, we meticulously designed the stimuli to balance the frequency of each strategy used in 10 social media posts. However, because we used real social media posts, the nature of real social media content meant that each post could incorporate several different strategies. Notably, messages targeting benefits and barriers in social media messages were more prevalent, while those addressing perceived susceptibility and perceived self-efficacy were less frequent. To mitigate this limitation, future research can conduct experiments that tightly manipulate the strategies used in social media posts and assess the impact of these posts on the audience's attitudes and health behavioral intentions.

Next, the composition of our sample poses limitations on the generalizability of our findings. For example, a large proportion of participants in our sample had undergraduate degrees. These differences raise concerns about the applicability of our findings to the broader population, given the influence of demographic factors such as education level on vaccination intention. Going forward, future studies should aim to recruit more diverse samples to better reflect the demographic diversity of the population, thereby increasing the generalizability and validity of the findings. Additionally, exploring how demographic factors interact with vaccination intentions could provide valuable insights into developing targeted interventions to address disparities in vaccination between different demographic groups. Future study needs to be based on more representative samples that are more reflective of various education levels, race/ethnicity, income levels. In addition, caution is needed to generalize our findings as our sample is based on the majority of the White population with more than a college degree who are making middle level of income. In future research, a comparison between the study sample and the general population demographics would help assess the generalizability of the findings more effectively.

Moreover, our study has shed light on the influence of different types of engagement behaviors on individuals' behavioral intentions, with a specific focus on the impact of “liking” a post. However, future research is necessary to explore the role of “sharing” and “commenting” on social media posts. Building upon this and previously published literature, future research should aim to identify which social media posts receive the most shares and whether this engagement behavior reflects the sharer's own attitudes or preferences or if they anticipate their own followers will appreciate the post, or both. Additionally, investigating the characteristics of posts that receive the most comments is important because it can help to determine if commenting signifies greater agreement or disagreement compared to other types of user engagement behaviors or if it is simply a result of specific individuals being more likely to engage in conversation. Therefore, future research should explore the characteristics of the audience that may be associated with sharing and commenting and investigate whether there is an interaction effect between audience characteristics and message attributes on people's engagement behaviors. In addition, future research should measure participants' intentions to leave pro-vaccine or anti-vaccine comments instead of measuring a composite one of commenting to provide a more nuanced and valuable understanding of the participants' vaccination intention.

In addition, it is imperative to acknowledge the diverse facets of user engagement in future research. This includes examining how individuals interact differently with pro-vaccine and anti-vaccine content on social media platforms, while also considering the intensity and duration of these interactions, as well as the roles played by different actors, such as content creators vs. commenters. A robust body of literature underscores the pivotal role of these parameters in shaping the effects of engagement on individuals' cognitions, intentions, and performance regarding health behaviors. Moving forward, future research should strive to incorporate these nuanced aspects to enrich our understanding of the dynamics surrounding user engagement and its impact on health behaviors.

Another consideration is the potential role of partisanship in shaping audience responses to social media content. Given the politicized nature of public health campaigns, individuals may use content creators' demographic characteristics as heuristics to infer their political stances, which could, in turn, influence engagement and behavioral intentions. However, our study did not directly measure partisanship or examine its effects on message reception. Future research should explore the intersection of political identity, source characteristics, and health messaging to better understand how these factors influence engagement and decision-making in public health contexts.

At the measurement level, we acknowledge that a test for common method bias (CMB) was not conducted. However, we performed Confirmatory Factor Analysis (CFA) to assess construct validity and tested multiple model specifications, ultimately selecting the best-fitting model, which supports the robustness of our findings. Additionally, while we did not conduct a multicollinearity test prior to SEM estimation, our correlation matrix indicates that the inter-variable correlations range between 0.3 and 0.7, suggesting that multicollinearity is unlikely to be a significant concern. Future research should incorporate explicit CMB assessments and formal multicollinearity diagnostics to further enhance methodological rigor.

Lastly, future research should explore how emerging social media technologies, such as artificial intelligence-driven content, virtual influencers, and immersive platforms, influence health communication and user engagement. As social media continues to evolve, user behaviors and preferences may shift, requiring new strategies to effectively disseminate health messages. Longitudinal studies could provide insights into how these changes impact the effectiveness of different messaging strategies over time. Additionally, future research could examine the role of personalized health communication, leveraging machine learning algorithms to tailor content based on individual risk perceptions and engagement patterns. Understanding these developments will help refine health communication strategies, ensuring they remain relevant and effective in an ever-changing digital landscape.

## Data Availability

The raw data supporting the conclusions of this article will be made available by the authors, without undue reservation.

## Appendix

**Appendix 1:** Correlations between all the variables.

	1	2	3	4	5	6	7	8	9	10	11	12	13	14	15	16	17	18	19	20
1	1																			
2	.37**	1																		
3	.10**	.09**	1																	
4	.17**	.16**	.63**	1																
5	.18**	.20**	.60**	.77**	1															
6	.10**	.08**	.66**	.61**	.66**	1														
7	.15**	.19**	.50**	.64**	.75**	.64**	1													
8	.21**	.20**	.59**	.71**	.82**	.71**	.71**	1												
9	−0.05	−0.03	−0.02	−0.02	−0.03	−0.002	−0.02	−0.01	1											
10	−0.03	0.02	−0.06	−0.04	−0.04	−0.03	−0.001	−0.03	−0.003	1										
11	−0.04	−0.02	0.04	0.02	0.01	−0.001	0.001	−0.01	.14**	−0.12**	1									
12	−0.02	−0.05	0.002	−0.004	−0.02	−0.02	−0.02	−0.01	0.02	−0.04	.30**	1								
13	−0.05	−0.04	0.04	0.01	0.02	0.05	0.01	0.03	−0.04	0.03	−0.04	.21**	1							
14	.20**	.27**	.51**	.53**	.59**	.52**	.54**	.69**	−0.03	0.002	−0.01	0.01	.06*	1						
15	.15**	.15**	.55**	.56**	.64**	.62**	.54**	.73**	−0.01	−0.02	−0.002	−0.001	0.04	.61**	1					
16	.14**	.18**	.56**	.56**	.61**	.58**	.54**	.72**	−0.03	−0.004	−0.018	0.01	0.05	.76**	.66**	1				
17	−0.03	−0.05	−0.02	−0.04	−0.03	−0.04	−0.02	−0.03	0.02	−0.06	0.05	.10**	0.03	−0.02	0.004	−0.02	1			
18	−0.02	−0.02	−0.05	−0.03	−0.02	−0.02	−0.02	−0.03	−0.01	−0.05	−0.02	−0.003	0.03	−0.04	−0.03	−0.03	−0.02	1		
19	0.001	0.04	0.03	0.03	0.01	−0.03	−0.01	0.02	−0.01	0.01	0.02	0.05	.06*	0.04	0.05	0.05	−0.10**	.31**	1	
20	−0.03	0.004	−0.07	−0.02	0.001	.09**	−0.03	−0.05	0.02	−0.05	0.05	.10**	−0.04	-.08**	.06*	−0.07**	.16**	0.03	−0.22**	1

1, vaccination intention; 2, the intention to persuade others to take the vaccine; 3, perceived susceptibility; 4, perceived severity; 5, perceived benefits; 6, perceived barriers; 7, perceived self-efficacy; 8, perceived cues to action; 9, message severity score; 10, message susceptibility score; 11, message benefits score; 12, message barriers score; 13, message self, efficacy score; 14, the number of favorites; 15, the number of shares; 16, the number of comments; 17, proportion of female content creators; 18, proportion of Black content creators; 19, proportion of politician content creators; 20, proportion of celebrity content creators.

* Correlation is significant at the 0.05 level (2-tailed).

** Correlation is significant at the 0.01 level (2-tailed).

## References

[1] Hadlington L Harkin LJ Kuss D Newman K Ryding FC. Perceptions of fake news, misinformation, and disinformation amid the COVID-19 pandemic: a qualitative exploration. Psychol Pop Media. (2023) 12(1):40. 10.1037/ppm0000387

[2] Gillani N Yuan A Saveski M Vosoughi S Roy D. Me, my echo chamber, and I. Proceedings of the 2018 World Wide Web Conference on World Wide Web—WWW ‘18 (2018). p. 823–831. 10.1145/3178876.3186130

[3] O’Brien HL Toms EG. What is user engagement? A conceptual framework for defining user engagement with technology. J Am Soc Inf Sci Technol. (2008) 59(6):938–955. 10.1002/asi.20801

[4] Alhabash S McAlister AR Lou C Hagerstrom A. From clicks to behaviors: the mediating effect of intentions to like, share, and comment on the relationship between message evaluations and offline behavioral intentions. J Interact Advert. (2015) 15(2):82–96. 10.1080/15252019.2015.1071677

[5] Benis A Seidmann A Ashkenazi S. Reasons for taking the COVID-19 vaccine by US social media users. Vaccines (Basel). (2021) 9(4):315. 10.3390/vaccines9040315 33805283 PMC8067223

[6] Xin M Luo S She R Chen X Li L Li L The impact of social media exposure and interpersonal discussion on intention of COVID-19 vaccination among nurses. Vaccines (Basel). (2021) 9(10):1204. 10.3390/vaccines9101204 34696312 PMC8537317

[7] Bruns A. Making sense of society through social media. Soc Media + Soc. (2015) 1(1):20–27. 10.1177/2056305115578679

[8] Gruzd A Staves K Wilk A. Connected scholars: examining the role of social media in research practices of faculty using the UTAUT model. Comput Human Behav. (2012) 28(6):2340–2350. 10.1016/j.chb.2012.07.004

[9] Kaplan AM Haenlein M. Users of the world, unite! the challenges and opportunities of social media. Bus Horiz. (2010) 53(1):59–68. 10.1016/j.bushor.2009.09.003

[10] Arceneaux P Dinu L. The social-mediated age of information: twitter and instagram as tools for information dissemination in higher education. New Media Soc. (2018) 20(11):4155–4176. 10.1177/1461444818768259

[11] O’Day EB Heimberg RG. Social media use, social anxiety, and loneliness: a systematic review. Comput Hum Behav Rep. (2021) 3:100070. 10.1016/j.chbr.2021.100070

[12] Naaman M Becker H Gravano L. Hip and trendy: characterizing emerging trends on twitter. J Am Soc Inf Sci Technol. (2011) 62(5):902–918. 10.1002/asi.21489

[13] Smith PJ Humiston SG Marcuse EK Zhao Z Dorell CG Howes C Parental delay or refusal of vaccine doses, childhood vaccination coverage at 24 months of age, and the health belief model. Public Health Rep. (2011) 126:135–146. 10.1177/00333549111260S215 21812176 PMC3113438

[14] Ellison NB Boyd D. Sociality through social network sites. In: Dutton WH, editor. The Oxford Handbook of Internet Studies. Oxford: Oxford University Press (2013). p. 151–172.

[15] Sawyer R Chen GM. The impact of new social media on intercultural adaptation. Intercult Commun Stud. (2012) 21:151–169.

[16] Melton CA Olusanya OA Ammar N Shaban-Nejad A. Public sentiment analysis and topic modeling regarding COVID-19 vaccines on the reddit social media platform: a call to action for strengthening vaccine confidence. J Infect Public Health. (2021) 14(10):1505–1512. 10.1016/j.jiph.2021.08.010 34426095 PMC8364208

[17] Liu PL. COVID-19 information on social media and preventive behaviors: managing the pandemic through personal responsibility. Soc Sci Med. (2021) 277:113928–113928. 10.1016/j.socscimed.2021.113928 33865093 PMC8040317

[18] Biella M Orrù G Ciacchini R Conversano C Marazziti D Gemignani A. Anti-vaccination attitude and vaccination intentions against COVID-19: a retrospective cross-sectional study investigating the role of media consumption. Clin Neuropsychiatry. (2023) 20(4):252. 10.36131/cnfioritieditore20230404 37791084 PMC10544246

[19] Martin LR Petrie KJ. Understanding the dimensions of anti-vaccination attitudes: the vaccination attitudes examination (VAX) scale. Ann Behav Med. (2017) 51(5):652–660. 10.1007/s12160-017-9888-y 28255934

[20] Chadwick A Vaccari C Kaiser J. The amplification of exaggerated and false news on social media: the roles of platform use, motivations, affect, and ideology. Am Behav Sci. (2025) 69(2):113–30. 10.1177/00027642221118264

[21] Huang Q Mao B Jia X Peng W. COVID-19 information overload mediated the effects of cross-channel information differences on health information elaboration. J Health Commun. (2023) 28(7):401–411. 10.1080/10810730.2023.2217097 37232168

[22] Jia X Ahn S Carcioppolo N. Measuring information overload and message fatigue toward COVID-19 prevention messages in USA and China. Health Promot Int. (2023) 38(3):daac003. 10.1093/heapro/daac003 35092282 PMC8807320

[23] Mao B Jia X Huang Q. How do information overload and message fatigue reduce information processing in the era of COVID-19? An ability–motivation approach. J Inf Sci. (2024) 50(5):1242–1254. 10.1177/01655515221118047

[24] Rosenstock IM. Historical origins of the health belief model. Health Educ Monogr. (1974) 2(4):328–335. 10.1177/109019817400200403 299611

[25] Carpenter CJ. A meta-analysis of the effectiveness of health belief model variables in predicting behavior. Health Commun. (2010) 25(8):661–669. 10.1080/10410236.2010.521906 21153982

[26] Rosenstock IM. Why people use health services. Milbank Q. (2005) 83(4):94–124. 10.1111/j.1468-0009.2005.00425.x

[27] Harrison JA Mullen PD Green LW. A meta-analysis of studies of the health belief model with adults. Health Educ Res. (1992) 7(1):107–116. 10.1093/her/7.1.107 10148735

[28] Janz NK Becker MH. The health belief model: a decade later. Health Educ Q. (1984) 11(1):1–47. 10.1177/109019818401100101 6392204

[29] Jones CL Jensen JD Scherr CL Brown NR Christy K Weaver J. The health belief model as an explanatory framework in communication research: exploring parallel, serial, and moderated mediation. Health Commun. (2015) 30(6):566–576. 10.1080/10410236.2013.873363 25010519 PMC4530978

[30] Zimmerman RS Vernberg D. Models of preventative health behavior: comparison, critique and meta-analysis. In: Albrecht G, editor. Advances in Medical Sociology, Health Behavior Models: A Reformulation. Greenwich, CT: JAI Press (1994). p. 45–67.

[31] Sohl SJ Moyer A. Tailored interventions to promote mammography screening: a meta-analytic review. Prev Med. (2007) 45:252–26. 10.1016/j.ypmed.2007.06.009 17643481 PMC2078327

[32] Bateman LB Hall AG Anderson WA Cherrington AL Helova A Judd S Exploring COVID-19 vaccine hesitancy among stakeholders in African American and latinx communities in the deep south through the lens of the health belief model. Am J Health Promot. (2021) 36(2):288–295. 10.1177/08901171211045038 34719985 PMC8770578

[33] Chen H Li X Gao J Liu X Mao Y Wang R Health belief model perspective on the control of COVID-19 vaccine hesitancy and the promotion of vaccination in China: web-based cross-sectional study. J Med Internet Res. (2021) 23(9):293–329. 10.2196/29329 PMC8425399 34280115

[34] Liu J Kassas B Lai J Kropp J Gao Z. Understanding the role of risk preferences and perceptions in vaccination decisions and post-vaccination behaviors among US households. Sci Rep. (2024) 14(1):3190. 10.1038/s41598-024-52408-6 38326338 PMC10850518

[35] Mercadante AR Law AV. Will they, or won't they? Examining patients’ vaccine intention for flu and COVID-19 using the health belief model. Res Soc Adm Pharm. (2021) 17(9):1596–1605. 10.1016/j.sapharm.2020.12.012 PMC7833824 33431259

[36] Jia X Ahn S Seelig MI Morgan SE. The role of health belief model constructs and content creator characteristics in social media engagement: insights from COVID-19 vaccine tweets. Healthcare. (2024) 12(18):18–45. 10.3390/healthcare12181845 PMC11431524 39337186

[37] Alhaimer R. The health belief model: evaluating governmental public health messages on social media aimed at preventing a COVID-19 epidemic in Kuwait. Cogent Bus Manage. (2022) 9(1):2031682. 10.1080/23311975.2022.2031682

[38] Zhang X Baker K Pember S Bissell K. Persuading me to eat healthy: a content analysis of YouTube public service announcements grounded in the health belief model. South Commun J. (2017) 82(1):38–51. 10.1080/1041794X.2016.1278259

[39] Tian S Cho SY Jia X Sun R Tsai WS. Antecedents and outcomes of generation Z consumers’ contrastive and assimilative upward comparisons with social media influencers. J Prod Brand Manage. (2023) 32(7):1046–62. 10.1108/JPBM-02-2022-3879

[40] Bonnevie E Gallegos-Jeffrey A Goldbarg J Byrd B Smyser J. Quantifying the rise of vaccine opposition on twitter during the COVID-19 pandemic. J Commun Healthc. (2021) 14(1):12–19. 10.1080/17538068.2020.1858222

[41] Durau J Diehl S Terlutter R. Motivate me to exercise with you: the effects of social media fitness influencers on users’ intentions to engage in physical activity and the role of user gender. Digit Health. (2022) 8:20552076221102769. 10.1177/20552076221102769 35615268 PMC9125114

[42] Kostygina G Tran H Binns S Szczypka G Emery S Vallone D Boosting health campaign reach and engagement through use of social media influencers and memes. Soc Media + Soc. (2020) 6(2):2056305120912475. 10.1177/2056305120912475

[43] Pavelko RL Myrick JG Verghese RS Hester JB. Public reactions to celebrity cancer disclosures via social media: implications for campaign message design and strategy. Health Educ J. (2017) 76(4):492–506. 10.1177/0017896917696122

[44] Giertz JN Weiger WH Törhönen M Hamari J. Content versus community focus in live streaming services: how to drive engagement in synchronous social media. J Serv Manag. (2022) 33(1):33–58. 10.1108/JOSM-12-2020-0439

[45] Schivinski B Christodoulides G Dabrowski D. Measuring consumers’ engagement with brand-related social-media content. J Advert Res. (2016) 56(1):64–80. 10.2501/JAR-2016-004

[46] Cavallo DN Tate DF Ries AV Brown JD DeVellis RF Ammerman AS. A social media-based physical activity intervention: a randomized controlled trial. Am J Prev Med. (2012) 43(5):527–532. 10.1016/j.amepre.2012.07.019 23079176 PMC3479432

[47] Draper CE Grobler L Micklesfield LK Norris SA. Impact of social norms and social support on diet, physical activity and sedentary behaviour of adolescents: a scoping review. Child. (2015) 41(5):654–667. 10.1111/cch.12241 25809525

[48] Majmundar A Chou C-P Cruz TB Unger JB. Relationship between social media engagement and e-cigarette policy support. Addict Behav Rep. (2018) 9:100155. 10.1016/j.abrep.2018.100155 31193757 PMC6542731

[49] Nelson LA Coston TD Cherrington AL Osborn CY. Patterns of user engagement with mobile- and web-delivered self-care interventions for adults with T2DM: a review of the literature. Curr Diab Rep. (2016) 16(7):1–20. 10.1007/s11892-016-0755-1 27255269 PMC5268129

[50] Cho M Schweickart T Haase A. Public engagement with nonprofit organizations on Facebook. Public Relat Rev. (2014) 40(3):565–567. 10.1016/j.pubrev.2014.01.008

[51] Yang Q Sangalang A Rooney M Maloney E Emery S Cappella JN. How is marijuana vaping portrayed on YouTube? Content, features, popularity and retransmission of vaping marijuana YouTube videos. J Health Commun. (2018) 23(4):360–369. 10.1080/10810730.2018.1448488 29533139 PMC12973428

[52] Devereux E Grimmer L Grimmer M. Consumer engagement on social media: evidence from small retailers. J Consum Behav. (2020) 19(2):151–159. 10.1002/cb.1800

[53] Berinsky AJ Huber GA Lenz GS. Evaluating online labor markets for experimental research: amazon.com's Mechanical Turk. Polit Anal. (2012) 20(3):351–368. 10.1093/pan/mpr057

[54] Buhrmester M Kwang T Gosling SD. Amazon’s mechanical Turk: a new source of inexpensive, yet high-quality, data? Perspect Psychol Sci. (2011) 6(1):3–5. 10.1177/1745691610393980 26162106

[55] Buhrmester M Talaifar S Gosling SD. An evaluation of Amazon’s mechanical turk, its rapid rise, and its effective use. Perspect Psychol Sci. (2018) 13(2):149–154. 10.1177/1745691617706516 29928846

[56] DeVerna MR Pierri F Truong BT Bollenbacher J Axelrod D Loynes N Covaxxy: a collection of English-language twitter posts about COVID-19 vaccines. Proceedings of the AAAI International Conference on web and Social media (ICWSM), Virtual Conference (2021).

[57] Al-Metwali BZ Al-Jumaili AA Al-Alag ZA Sorofman B. Exploring the acceptance of COVID-19 vaccine among healthcare workers and general population using health belief model. J Eval Clin Pract. (2021) 27(5):1112–1122. 10.1111/jep.13581 33960582 PMC8242385

[58] Jia X Ahn S. Psychometric analysis of items evaluating health belief model constructs in social Media posts: application of rasch measurement model. Behav Sci. (2025) 15(2):204. 10.3390/bs15020204 40001834 PMC11851386

[59] Wong MC Wong EL Huang J Cheung AW Law K Chong MK Acceptance of the COVID-19 vaccine based on the health belief model: a population-based survey in Hong Kong. Vaccine. (2021) 39(7):1148–1156. 10.1016/j.vaccine.2020.12.083 33461834 PMC7832076

[60] Yu Y Lau JT She R Chen X Li L Li L Prevalence and associated factors of intention of COVID-19 vaccination among healthcare workers in China: application of the health belief model. Hum Vaccin Immunother. (2021) 17(9):2894–2902. 10.1080/21645515.2021.1909327 33877955 PMC8381834

[61] Ibrahim B Aljarah A Hayat DT Lahuerta-Otero E. Like, comment and share: examining the effect of firm-created content and user-generated content on consumer engagement. Leisure/Loisir. (2022) 46(4):599–622. 10.1080/14927713.2022.2054458

[62] Hossain MB Alam MZ Islam MS Sultan S Faysal MM Rima S Health belief model, theory of planned behavior, or psychological antecedents: what predicts COVID-19 vaccine hesitancy better among the Bangladeshi adults? Front Public Health. (2021) 9:711066. 10.3389/fpubh.2021.711066 34490193 PMC8418098

[63] Preacher KJ Coffman DL. Computing power and minimum sample size for RMSEA. (2006). Available at: http://www.quantpsy.org/rmsea/rmsea.htm (Accessed June 12, 2024).

[64] Muthén LK Muthén BO. Mplus User’s Guide. Eighth Edition. Los Angeles, CA: Muthén & Muthén (1998–2017).

[65] Chapman S. Does celebrity involvement in public health campaigns deliver long term benefit? Yes. Br Med J. (2012) 345:e6364. 10.1136/bmj.e6364 23015036

[66] Veale HJ Sacks-Davis R Weaver E Pedrana AE Stoové MA Hellard ME. The use of social networking platforms for sexual health promotion: identifying key strategies for successful user engagement. BMC Public Health. (2015) 15(1):1–11. 10.1186/s12889-015-1396-z 25884461 PMC4340797

[67] Zhang Y Xia T Huang L Yin M Sun M Huang J Factors influencing user engagement of health information disseminated by Chinese provincial centers for disease control and prevention on WeChat: observational study. JMIR Mhealth Uhealth. (2019) 7(6):e12245. 10.2196/12245 31250833 PMC6620885

[68] Marsh D Hart PT Tindall K. Celebrity politics: the politics of the late modernity? Political Stud Rev. (2010) 8(3):322–340. 10.1111/j.1478-9302.2010.00215.x

[69] Wheeler M. Celebrity Politics. Cambridge: Polity (2013).

[70] Manning N Penfold-Mounce R Loader BD Vromen A Xenos M. Politicians, celebrities and social media: a case of informalisation? J Youth Stud. (2017) 20(2):127–144. 10.1080/13676261.2016.1206867

[71] Massey PM Kearney MD Hauer MK Selvan P Koku E Leader AE. Dimensions of misinformation about the HPV vaccine on Instagram: content and network analysis of social media characteristics. J Med Internet Res. (2020) 22(12):e21451. 10.2196/21451 33270038 PMC7746500

[72] Guidry JP Carlyle KE LaRose JG Perrin P Messner M Ryan M. Using the health belief model to analyze Instagram posts about Zika for public health communications. Emerg Infect Dis. (2019) 25(1):179. 10.3201/eid2501.180824 30561302 PMC6302587

[73] Badr H Zhang X Oluyomi A Woodard LD Adepoju OE Raza SA Overcoming COVID-19 vaccine hesitancy: insights from an online population-based survey in the United States. Vaccines (Basel). (2021) 9(10):1100. 10.3390/vaccines9101100 34696208 PMC8539129

[74] Le CN Nguyen UTT Do DTH. Predictors of COVID-19 vaccine acceptability among health professions students in Vietnam. BMC Public Health. (2022) 22(1):1–12. 10.1186/s12889-021-12274-7 35484522 PMC9047623

[75] Toth-Manikowski SM Swirsky ES Gandhi R Piscitello G. COVID-19 vaccination hesitancy among health care workers, communication, and policy-making. Am J Infect Control. (2022) 50(1):20–25. 10.1016/j.ajic.2021.10.004 34653527 PMC8511871

[76] Wang Y Duan L Li M Wang J Yang J Song C COVID-19 vaccine hesitancy and associated factors among diabetes patients: a cross-sectional survey in Changzhi, Shanxi, China. Vaccines (Basel). (2022) 10(1):129. 10.3390/vaccines10010129 35062790 PMC8778010

[77] Guidry J Laestadius LI Vraga EK Miller CA Perrin PB Burton CW Willingness to get the COVID-19 vaccine with and without emergency use authorization. Am J Infect Control. (2021) 49(2):137–142. 10.1016/j.ajic.2020.11.018 33227323 PMC7677682

[78] Azer J Blasco-Arcas L Harrigan P. #COVID-19: forms and drivers of social media users’ engagement behavior toward a global crisis. J Bus Res. (2021) 135:99–111. 10.1016/j.jbusres.2021.06.030 36540310 PMC9754676

[79] Grover P Kar AK. User engagement for mobile payment service providers–introducing the social media engagement model. J Retail Consum Serv. (2020) 53:101718. 10.1016/j.jretconser.2018.12.002

[80] Maher CA Lewis LK Ferrar K Marshall S De Bourdeaudhuij I Vandelanotte C. Are health behavior change interventions that use online social networks effective? A systematic review. J Med Internet Res. (2014) 16(2):e40. 10.2196/jmir.2952 24550083 PMC3936265

